# DOES RACE MATTER IN HEALTH MESSAGING? IMPACT OF RACE CONCORDANCE ON PALLIATIVE CARE KNOWLEDGE SCORE

**DOI:** 10.1093/geroni/igac059.849

**Published:** 2022-12-20

**Authors:** Deborah Hoe, Mutian Zhang, Susan Enguidanos

**Affiliations:** University of Southern California, Los Angeles, California, United States; University of Southern California, Los Angele, California, United States; University of Southern California, Los Angeles, California, United States

## Abstract

Palliative care (PC) programs have expanded rapidly across the United States in the last decade. However, there remains a wide disparity in access to PC among ethnic minorities. Therefore, it is necessary and critical to develop specific health-related educational tools targeted for the vastly diverse, community-based older adult population. This study seeks to investigate how race concordance in health-related messaging might affect changes in PC knowledge scores among Black and White, English-speaking, community-based adults aged 50 and older. We recruited 406 Black and White adults online and from community-based sites. Sixty-four percent were shown race concordant patient stories, the remainder were shown videos of opposite race patients. Pretest-posttest study design was employed and participants completed a 20-question survey about PC knowledge, intent to seek PC, and perceptions about the videos. Regression analysis was conducted to test if race concordance improved knowledge after viewing patient stories.PC knowledge score (max=13) improved from an average of 5.70 to 11.23 (t=23.2, p< 0.001). Participants that watched same-race videos for both Black and White registered greater significant score increases (White:1.26±0.36 points, p=0.001; Black:0.95±0.38 points, p< 0.05) compared with those who watched different-race videos. Further, intent to enroll oneself in PC revealed statistically significant relationships in all groups except among White participants who viewed different-race videos (p=0.157, Fisher’s exact test, two-tailed). This study suggests that while PC role model stories improved PC knowledge across the board, the improvement is greater in race concordant groups, which also has implications for changes in intent to enroll in PC.

